# Identifying Natural Substrates for Dipeptidyl Peptidases 8 and 9 Using Terminal Amine Isotopic Labeling of Substrates (TAILS) Reveals *in Vivo* Roles in Cellular Homeostasis and Energy Metabolism*[Fn FN3]^♦^

**DOI:** 10.1074/jbc.M112.445841

**Published:** 2013-03-21

**Authors:** Claire H. Wilson, Dono Indarto, Alain Doucet, Lisa D. Pogson, Melissa R. Pitman, Kym McNicholas, R. Ian Menz, Christopher M. Overall, Catherine A. Abbott

**Affiliations:** From the ‡ School of Biological Sciences and; ¶ Flinders Centre for Innovation in Cancer, Flinders University, Adelaide, South Australia 5001, Australia and; the § Departments of Biochemistry and Molecular Biology and Oral Biological and Medical Sciences, Centre for Blood Research and Faculty Dentistry, University of British Columbia, Vancouver, British Columbia V6T 1Z3, Canada

**Keywords:** Aminopeptidase, Cell Metabolism, Energy Metabolism, Enzymes, Proteomics, DP8/DPP8, DP9/DPP9, Adenylate Kinase, Calreticulin, Dipeptidyl Peptidase

## Abstract

Dipeptidyl peptidases (DP) 8 and 9 are homologous, cytoplasmic N-terminal post-proline-cleaving enzymes that are anti-targets for the development of DP4 (DPPIV/CD26) inhibitors for treating type II diabetes. To date, DP8 and DP9 have been implicated in immune responses and cancer biology, but their pathophysiological functions and substrate repertoire remain unknown. This study utilizes terminal amine isotopic labeling of substrates (TAILS), an N-terminal positional proteomic approach, for the discovery of *in vivo* DP8 and DP9 substrates. *In vivo* roles for DP8 and DP9 in cellular metabolism and homeostasis were revealed via the identification of more than 29 candidate natural substrates and pathways affected by DP8/DP9 overexpression. Cleavage of 14 substrates was investigated *in vitro*; 9/14 substrates for both DP8 and DP9 were confirmed by MALDI-TOF MS, including two of high confidence, calreticulin and adenylate kinase 2. Adenylate kinase 2 plays key roles in cellular energy and nucleotide homeostasis. These results demonstrate remarkable *in vivo* substrate overlap between DP8/DP9, suggesting compensatory roles for these enzymes. This work provides the first global investigation into DP8 and DP9 substrates, providing a number of leads for future investigations into the biological roles and significance of DP8 and DP9 in human health and disease.

## Teaser

**Background:** Biological roles for intracellular dipeptidyl peptidases 8 and 9 are unknown.

**Results:** By degradomics, 29 new *in vivo* substrates were identified (nine validated) for DP8/DP9, including adenylate kinase 2 and calreticulin.

**Conclusion:** These substrates indicate roles for DP8 and DP9 in metabolism and energy homeostasis.

**Significance:** Being the first proteomics screen for DP8/DP9 substrates, unexpected new cellular roles were revealed.

## Introduction

Dipeptidyl peptidase 8 (DP8/DPP8/dipeptidyl peptidase IV-related protein 1 (DPRP-1)) and DP9 (DPP9/DPRP-2) are highly conserved and ubiquitously expressed intracellular exopeptidases of the serine protease SC clan S9b subfamily (which includes (DPP4/DPIV/DPPIV/CD26 EC 3.4.14.5)) that share 61% identity at the amino acid level in humans (1–4). Studies utilizing nonselective DP^7^ inhibitors (5) and more selective DP8/DP9 inhibitors (6–8) have suggested an important immunological role for DP8/DP9. DP8/DP9 have also been implicated in the allergic response of the lung (9) and inflammatory bowel disorders (10). *In vitro* studies have demonstrated nonenzymatic roles for DP8 and DP9 in cell migration, proliferation, and apoptosis (11). In cancer, increased DP8 mRNA has been found in chronic lymphocytic leukemia (12) and DP9 mRNA in testicular cancer (3), and increased levels of DP8/DP9 mRNA, protein, and enzymatic activity have been observed in human meningiomas (13). Ubiquitous but differential expression of DP8/DP9 has been observed in breast and ovarian carcinoma cell lines (14), and a study has identified DP8/DP9 as survival factors for the Ewing sarcoma family of tumors (15). Despite these findings, the mechanism(s) of DP8/DP9 function in these events has yet to be identified, and their exact biological roles remain unknown. Uncovering protease substrates greatly assists in revealing the functions of proteases *in vivo* and their significance in pathophysiological processes (16, 17).

*In vitro*, both DP8 and DP9 cleave the well known DP4 substrates neuropeptide Y(1–36), glucagon-like peptide-1(7–36), glucagon-like peptide-2(1–33), and peptide YY(1–36) (18, 19). *In vitro,* DP8 cleavage of chemokine stromally derived factor 1 (CXCL12)-α/β, interferon-γ-inducible protein, and interferon-inducible T cell α-chemoattractant, also known DP4 substrates, has been demonstrated (20). However, as DP8 and DP9 are intracellular enzymes, it is unlikely that these secreted substrates will be of physiological relevance *in vivo*. Clues for their *in vivo* roles come from studies blocking DP8/DP9 or DPIV expression, which leads to neuropeptide Y-driven cell death within the Ewing sarcoma family of tumors cells (15). To date, the first and only *in vivo* substrate identified for DP9 is the antigenic peptide renal ubiquitous-1(34–42) with DP9 proteolysis preventing major histocompatibility complex class I cell surface antigen presentation (21). Besides these initial studies, no comprehensive effort has been made to identify the *in vivo* substrates of DP8 and DP9 on a system-wide scale.

This study used a positional N-terminal proteomics approach, terminal amine isotopic labeling of substrates (TAILS), to identify the substrate degradome of DP8 and DP9. TAILS is focused around the isolation of protein and peptide N termini for proteomic identification of neo-N termini resulting from proteolytic events (22). This method was recently used to detect cleavage events *in vivo* in inflamed mouse skin (23). Stable human ovarian cancer (SKOV3) cell lines expressing enzyme-active and catalytically inactive forms of DP8 and DP9 were generated, and their cytoplasmic proteomes were isolated and analyzed by TAILS. A number of candidate substrates were identified and confirmed, including two of biological interest, calreticulin and adenylate kinase 2. This work reveals the involvement of DP8 and DP9 in cellular energy homeostasis pathways in this ovarian cancer cell line.

## EXPERIMENTAL PROCEDURES

All chemicals were purchased from Sigma unless stated otherwise.

### 

#### 

##### Stable Cell Culture and Flow Cytometry

SKOV3 cells were maintained (14) with G418 addition (500 μg/ml) to stable transfectants. FuGENE® 6 (Roche Diagnostics) was used to stably transfect cells with constructs of pEGFPN1 (Clontech) alone or with wild-type human DP8_(882 aa)_ and DP9_(863 aa, short form)_ (where aa is amino acid) or catalytically inactive mutants DP8(S739A) and DP9(S729A) (11). Clonal cell lines were generated by single cell sorting of transfected parental cells using a FACSAria (Pharmingen) with initial supplementation of growth medium with 0.5× hybridoma fusion cloning supplement (Roche Diagnostics) and gentamicin (16 mg/ml). Stable EGFP-expressing transfectants were monitored using a FACScan (Pharmingen).

##### Isolation of Cytoplasmic Proteomes

Cells were grown to confluence in three T175 flasks, washed with PBS (three times, 10 ml) to remove serum proteins, and then incubated for 3 h in 15 ml of phenol red-free, serum-free DMEM (Invitrogen). Cells were washed with ice-cold PBS, detached with 0.2% (w/v) EDTA/PBS, and then resuspended in ice-cold PBS. Cells (0.5–2 × 10^7^) pooled from three flasks were pelleted by centrifugation (1500 × *g*, 5 min), resuspended in homogenization buffer (50 mm HEPES, pH 7.2, 200 mm NaCl, 10 mm CaCl_2_) containing protease inhibitors (0.2 mm PMSF, 10 μm E64, 1 μm pepstatin A, and 1 mm EDTA), and then gently lysed by nitrogen cavitation (800 p.s.i., 30 min on ice) using a Parr Cell Disruptor (Parr Instrument Co.). Lysates were clarified by centrifugation (1500 × *g*, 10 min, and 4 °C), and then supernatants were subjected to ultracentrifugation (type 70.1 Ti rotor, 100,000 × *g*, 60 min at 4 °C; Beckman Coulter Ultracentrifuge; Palo Alto, CA) for separation of membrane and cytosolic fractions. Cytosolic fractions were further clarified by additional ultracentrifugation (100,000 × *g*, 30 min, 4 °C). Protein concentrations were determined by micro-Bradford assay (Bio-Rad). Aliquots were made and stored at −70 °C until TAILS proteome analysis.

##### TAILS

TAILS was performed using dimethylation labeling of primary amines of protein N termini and ϵ-amino acids of lysine side chains as described (22). In brief, equal quantities of cytosolic proteome (supplemental Table S1) from enzyme-active and catalytically inactive cell lines (both DP8 and DP9) were concentrated and purified by 12% (v/v final) TCA precipitation. Protein denaturation, reduction, and alkylation of cysteine residues was performed as described previously (22). Samples were isotopically labeled by dimethylation using formaldehyde as described previously (22) (supplemental Table S1). Residual formaldehyde was quenched; then heavy and light isotopically labeled samples were combined, concentrated, and purified by acetone precipitation and tryptically digested, and then labeled peptides were enriched by a negative selection step using a dendritic polyglycerol aldehyde polymer as described (22). Blocked N-terminal peptides (unbound) were physically separated from the polymer-captured peptides via 10-kDa Microcon centrifugation (Millipore, Bedford, MA) (22). Sample and wash flow-throughs were combined and then prepared for off-line strong cation exchange-HPLC fractionation or desalting (supplemental Table S1). Three biological replicates for both DP8 and DP9 were subjected to the TAILS process in three independent experiments.

##### Off-line Strong Cation Exchange-HPLC Fractionation and Peptide Desalting

Desalting and pre-fractionation of samples (∼100 μg) by strong cation exchange-HPLC, prior to LC-MS/MS analysis, was performed as described (24). N-terminal peptides not fractionated by strong cation exchange-HPLC (supplemental Table S1) were desalted using reverse phase-solid phase extraction via Sep-Pak® C_18_ column (Waters, Milford, MA) according to the manufacturer's recommendations.

##### In-line Liquid Chromatography and Tandem-Mass Spectrometry

Peptide samples were analyzed by in-line reverse-phase nanospray LC-MS/MS using a C18 column (150-mm × 100-μm column at a flow rate of 100–200 nl min^−1^) coupled to a quadrupole time-of-flight QStar XL hybrid electrospray ionization mass spectrometer (Applied Biosystems/MDS-Sciex, Concord, Ontario, Canada) or a QStar Pulsar mass spectrometer (Applied Biosystems/MDS-Sciex, MDS-Sciex, Concord, Ontario, Canada). Samples were loaded, eluted, and separated on the C18 column, and MS data were acquired automatically using Analyst QS version 1.1 software (Applied Biosystems/MDS-Sciex, Concord, ON, Canada) as described (25).

##### Mass Spectrometry Data Analysis

MS peak lists were searched by MASCOT (version 2.2, Matrix Science, London, UK) against the human International Protein Index (IPI) database (version 3.16, 62,322 entries, release date April, 2006). MASCOT searches of MS data were performed separately for heavy- and light-labeled peptides. Searches were performed using the following modifications: fixed carbamidomethylation of cysteines (+57.021 Da (Cys)), fixed heavy lysine (+34.0631 Da (Lys)), or light lysine (+28.0311 Da (Lys)); variable methionine oxidation (+15.995 Da (Met)), and fixed and variable modifications of N termini with heavy formaldehyde (+34.0641 Da (N termini)), light formaldehyde (+28.0311 Da (N termini)), and acetylation (+42.011 Da (N termini)). The additional search criteria used were as follows: semi-ArgC cleavage specificity with up to three missed cleavages; a monoisotopic mass error window for the parent ion of 0.4 to 0.6 Da; peptide mass tolerance of 0.4 Da for MS/MS fragment ions; and the scoring scheme ESI-QUAD-TOF. Allowed peptide charge states were 1^+^, 2^+^, and 3^+^. Search results were analyzed using the Trans-Proteomic Pipeline (version 4.0 JETSTREAM revision 2, Build 200807011544 (MinGW)) (26) with PeptideProphet (27) sensitivity-error rate analysis. Quantification of the ratio of heavy to light isotopically labeled peptides was achieved by using ASAPRatio (28) software. ASAPRatios were manually checked and edited, and CLIPPER was used to identify statistically significant changes in cleaved neo-N peptides (29).

DP8 and DP9 are exopeptidases with a strong preference for cleavage of N-terminal dipeptides mainly after a Pro residue in the P1 position (NH_2_-P2-P1-P1′-P2′-). Because of this strict canonical specificity, datasets were also manually parsed to identify all peptides with a cleaved or noncleaved Pro residue in what would be the P1 position of a DP8/DP9 substrate. Pairs of peptides that differed in length by two amino acids at their N termini, but did not contain a Pro in P1, were also selected as candidate substrates.

Additional substrates and proteins that are altered (in expression or non-DP8/DP9 proteolysis) via DP8/DP9-affected pathways were identified from quantitative analysis of heavy/light (protease/control) isotope-labeled peptide abundance ratios. Only peptides identified with ≥95% confidence (PeptideProphet) were used for quantitative analysis. ASAPRatios were normalized by recentering the raw datasets around a median of one and then applying a natural logarithm transformation. This enabled the exclusion of obvious outliers and established a normal range for determining significance by using means and S.D. of the normalized data to determine 90% confidence intervals of the nontransformed data. This was achieved using the R statistical package. High and low ratio peptides were considered to be either substrates of DP8/DP9 or derived from proteins that were either differentially expressed or processed by an alternative protease to DP8/DP9.

##### Functional Annotation and Pathway Mapping

UniProt Knowledge Base (UniProtKB) accession numbers were mapped to protein IPI numbers. Functional annotation and biological pathway information were obtained from UniProtKB entries or by using the database for annotation, visualization and integrated discovery (DAVID), version 6.7 (30) (david.abcc.ncifcrf.gov). The TopFIND knowledge base was used to obtain functional insights to the cleaved substrates (31).

##### DP-specific Enzyme Assay

Diprolyl peptidase enzymatic activity was assayed using 0.5 mm H-Ala-Pro-*p*-nitroanilide (Bachem, Bubendorf, Switzerland) as described previously (14). Enzyme activity was expressed as milliunits/mg of protein, where 1 unit of activity is defined as the amount of enzyme that cleaves 1 μmol of substrate per min under the given assay conditions.

##### Western Blot Analysis

Protein extraction, SDS-PAGE, and Western blotting were performed as described previously (14) using primary antibodies for DP8 (RP1-DP8, Triple Point Biologics Inc.), DP9 (RP1-DP9, Triple Point Biologics Inc.), β-actin (ab8227, Abcam, Cambridge, UK, or 011M4812, Sigma), calreticulin (sc-6468, Santa Cruz Biotechnology Inc., Santa Cruz, CA), and adenylate kinase 2 (ab37594, Abcam).

##### In Vitro Validation of DP8 and DP9 Substrate Cleavage with MALDI-TOF MS Analysis

Recombinant human DP8_(882aa)_ and DP9_(892aa)_ were expressed and purified as described previously (20, 32). N-terminal peptides of 14 proteins identified by TAILS to be candidate substrates (Table 3) were synthesized to >95% purity by Genscript (Piscataway, NJ) or GL Biochem Ltd. (Shanghai, China). Peptides (10 μm) were incubated with 1.7 milliunits of recombinant DP8 or DP9 in assay buffer: 50 mm Tris, 100 mm NaCl, pH 8.0, at 37 °C for up to 24 h. Calreticulin and adenylate kinase 2 were also incubated in assay buffer alone or with DP8/DP9 in the presence of 10 μm of the DP inhibitor, Val-Boro-Pro (PT-100/Talbostat; obtained from Dr. Jonathon Cheng (Fox Chase Cancer Centre, Philadelphia) with approval from DARA BioSciences Inc., Raleigh, NC). At time points of 0, 1, 4, and 24 h, 5 μl of each reaction was removed and stopped by the addition of 1% TFA. Peptides were desalted and cleaned by OMIX® C18 tip (Varian, Inc., Palo Alto, CA). Eluates were mixed 1:1 with α-cyano-3-hydroxycinnamic acid matrix solution (1% (w/v) α-cyano-3-hydroxycinnamic acid, 49.5% acetonitrile, 49.5% ethanol, 0.001% TFA) and spotted onto a standard stainless steel MALDI sample plate, and then masses of intact peptides and DP8/DP9 cleavage products were obtained by MALDI-TOF MS analysis performed on a Waters Micromass® M@ALDI (Waters Micromass, Manchester, UK) or a Bruker Autoflex III MALDI MS/MS (Bruker, Billerica, MA). All cleavage experiments with MALDI-TOF analysis were performed in triplicate. MS spectra were processed using MassLynx 4.0 (Waters) software package.

##### Accession Information

All LC-MS/MS data (.wiff files) associated with this study may be downloaded from ProteomeCommons.org Tranche using the following hash: YRRnIo2jM9SRJ7vSBk7OG65e/3cVbIQ7ksECUIT97Doxwfg1YAGWJ5eHBqzMXog/IQFcGWzlS08gVKP/RqN+E1KLoVwAAAAAAAAYRg.

## RESULTS

### 

#### 

##### Generating Stable Clonal Cell Lines of Wild-type and Mutant DP8 and DP9

**FIGURE 1.**
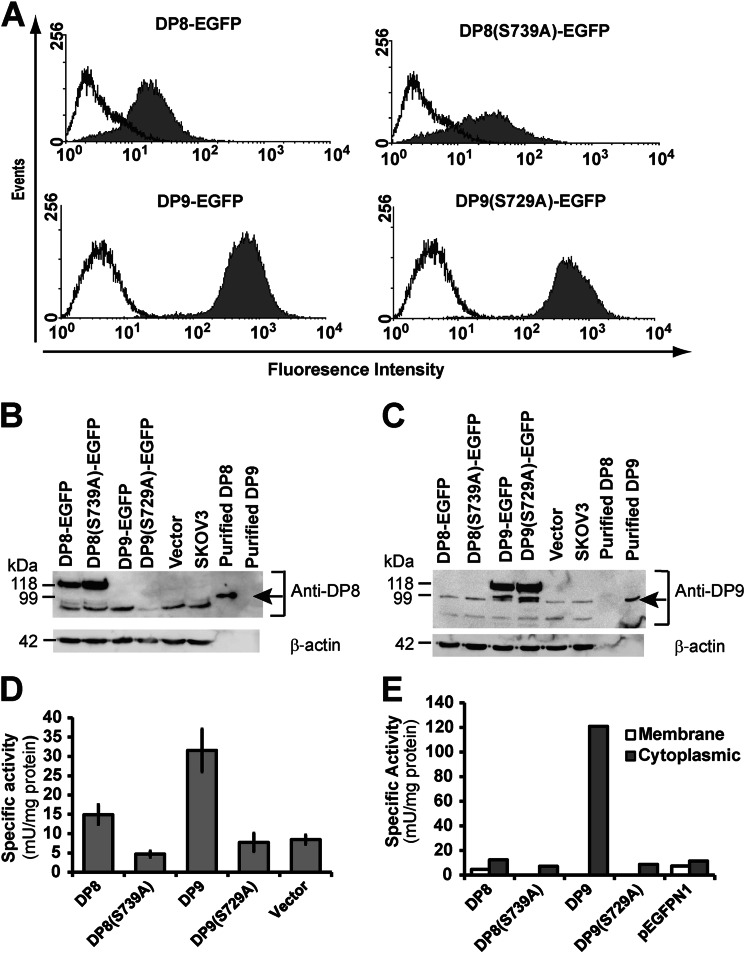
**Characterization of stable wild-type and mutant DP8 and DP9 SKOV3 cell lines.** *A,* cells transfected with wild-type (DP8-EGFP and DP9-EGFP) or mutant (DP8(S739)-EGFP and DP9(S729A)-EGFP) constructs (*filled histograms*) or nontransfected cells (*open histograms*) were analyzed by fluorescent flow cytometry on a FACScan. *B* and *C*, cell lysates (50 μg) from DP8-EGFP, DP8(S739A)-EGFP, DP9-EGFP, DP9(S729A)-EGFP, vector-transfected, and nontransfected SKOV3 cells were analyzed by 8% (w/v) SDS-PAGE and immunoblotting for detection of DP8 (*B*) or DP9 (*C*). Recombinant purified DP8 and DP9 were included as controls and are indicated by *arrows*. For a loading control, β-actin was detected. *D* and *E,* specific activity against the synthetic DP substrate H-Ala-Pro-*p*-nitroanilide (0.5 mm) was determined in whole cells (*D*) and in membrane and soluble fractions (*E*). Values in *D* are expressed as means ± S.E. (*n* = 10). Values in *E* are from a single experiment.

SKOV3 cells were stably transfected with constructs encoding enzymes DP8 and DP9, with a size of 882 and 863 residues, respectively, including the N-terminal β-propeller and C-terminal α/β-hydrolase domains. Clonal cell lines for both DP8 and DP9 were selected for equal expression of the active and inactive version of each enzyme in fusion with the fluorescent EGFP protein (Fig. 1*A*). DP8- and DP9-EGFP proteins were visualized by immunoblot (∼118 kDa in mobility) confirming the heterologous expression of active and inactive proteases (Fig. 1*B*). All cell lines were found to express basal levels of endogenous DP8 and DP9 protein (∼98 kDa in mobility) (Fig. 1*B*) that may contribute to background proteolysis and affect the discovery of substrates by TAILS. DP activity increased in the wild-type DP8- and DP9-transfected SKOV3 cells when compared with the catalytically inactive DP8(S739A), DP9(S729A), and vector-transfected controls (Fig. 1*C*). Enzymatic assays of fractionated clonal cell lines confirmed that this increase in DP activity is due to the overexpression of cytosolic DP8/DP9 (Fig. 1, *D* and *E*).

##### TAILS N-terminome Analysis of DP8 and DP9 Stable Cytoplasmic Proteomes

**FIGURE 2.**
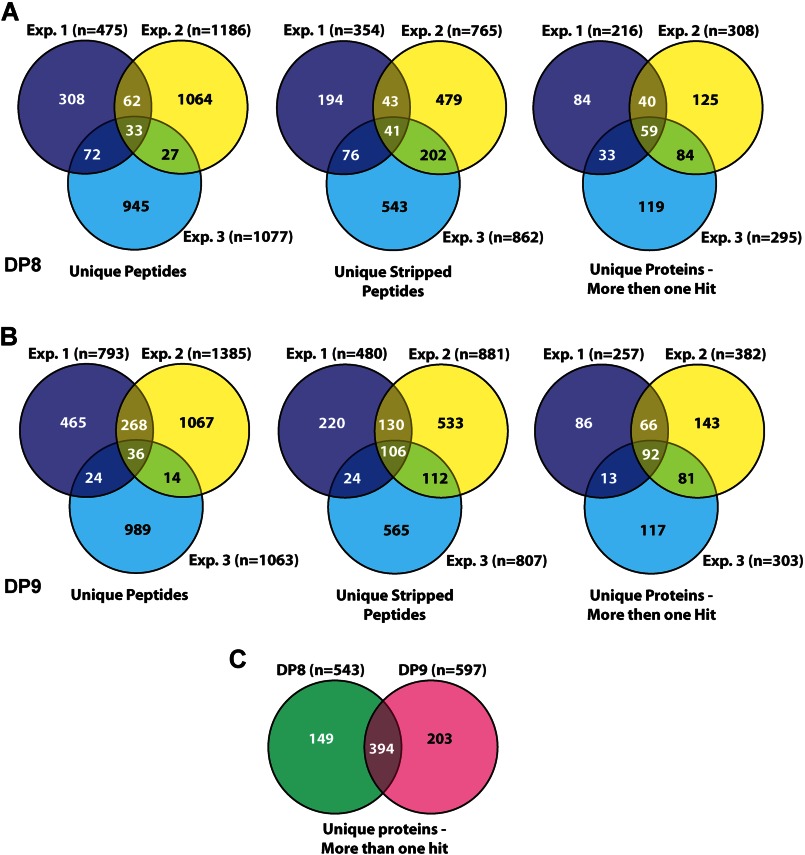
**Summary of total peptides and proteins identified by TAILS analysis.** *A* and *B,* three-way Venn diagrams of the number of unique peptides, unique stripped peptides, and unique proteins (identified by more then one spectra) identified by MASCOT searches in experiments (Exp.) 1, Exp. 2, and Exp. 3 for DP8 (*A*) and DP9 (*B*). *C,* two-way Venn diagram of unique proteins (identified by more then one hit) identified from all datasets for DP8 and DP9. The intersection of *C* displays the number of overlapping proteins identified in both the DP8 and DP9 datasets. The number of unique peptides for each experiment takes into account all possible modifications of a given peptide, including variable oxidation of methionine residues, although the number of unique stripped peptides for each experiment refers to unique peptides after the removal (stripping) of all possible modifications. The total number of unique proteins excludes any “single hit” proteins, *i.e.* a protein that is not identified by any other peptide/spectra in the dataset. All peptides were identified with ≥95% confidence according to PeptideProphet. All MASCOT searches were performed against the human IPI database (version 3.16, 62,322 entries, release date 4/2006).

TAILS was performed on three isolations of cytoplasmic proteomes from both wild-type and catalytically inactive DP8- and DP9-stable SKOV3 cell lines. The total number of unique peptides and proteins identified within each experiment was determined (Fig. 2, *A* and *B*). Each TAILS experiment yielded 200–400 unique proteins that were identified from more than one N-terminal peptide occurrence, *i.e.* peptide/spectra (Fig. 2, *A* and *B*). The union of each DP8 TAILS experiment yielded 59 unique proteins (Fig. 2*A*), whereas the union of each DP9 TAILS experiment yielded 92 unique proteins (Fig. 2*B*). From all three experiments, a total of 543 unique proteins were identified for DP8, and 597 were identified for DP9, of which 394 proteins were common to both DP8 and DP9 (Fig. 2*C*).

##### Identification of Candidate DP8/DP9 Substrates by Parsing TAILS Data

**FIGURE 3.**
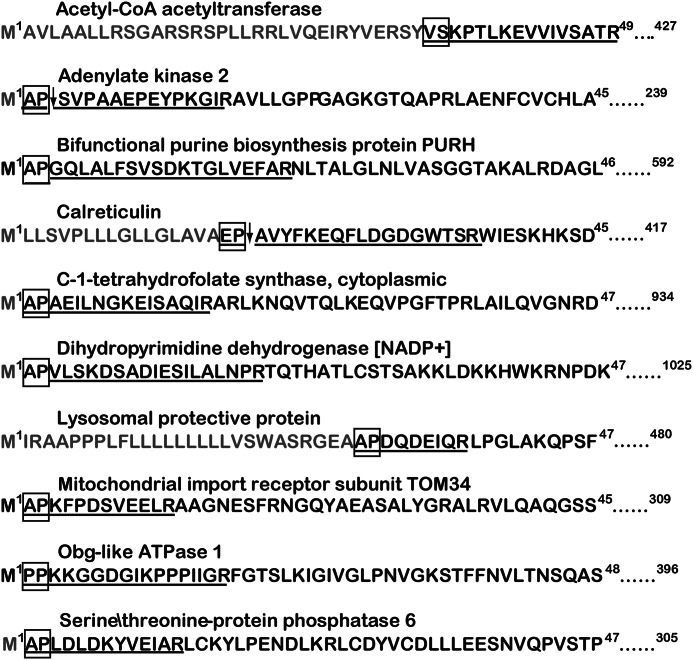
**Peptides of several candidate substrates identified by TAILS.** Peptides identified by MS/MS are indicated by *bold underlined letters. Light colored letters* indicate precursor peptides of mature proteins or their initiator Met residues that are known to be removed as annotated in UniProtKB. The dipeptide residues identified as being cleaved, or as having the potential for cleavage, are outlined by a *box*. Where two peptides were identified by MS/MS as being the noncleaved DP8/DP9 precursor peptide (N-terminal dipeptide intact) and the DP8/DP9 cleavage product peptide (N-terminal dipeptide removed), an *arrow* is used to indicate the site of DP8/DP9 proteolysis. Peptides for C-1-tetrahydrofolate synthase and cytoplasmic and lysosomal protective proteins were identified from manual parsing of the DP8 dataset. All other proteins were identified in both DP8 and DP9 datasets. The peptides corresponding to three of the potential cleavage sites indicated for fructose-bisphosphate aldolase A were only identified from manual parsing of the DP9 dataset (see Table 1). Full-length protein sequences of the above and other potential substrates can be found in supplemental Figs. S2 and S3.

A total of 22 and 21 proteins were identified by parsing for potential DP8 and DP9 substrates, respectively. Of these, 8/22 and 7/21 candidate substrates were uniquely identified for DP8 and DP9, respectively (Table 1). Most candidate substrates are localized to the cytoplasm or intracellular organelles (Table 1). Adenylate kinase 2 and calreticulin were two predominant proteins for which the most cleaved and noncleaved peptides were identified for both DP8 and DP9 (Table 1). Approximately half of the peptides identified with the potential for DP8/DP9 cleavage were located at the N termini of the mature proteins as annotated in UniProtKB (Fig. 3, Table 1, and supplemental Figs. S2 and S3). Functional annotation and pathway mapping revealed the involvement of candidate substrates in biochemical pathways relating to lysosomal processes, carbohydrate metabolism, and nucleotide metabolism and synthesis (Table 1; supplemental Tables S6 and S7).

**TABLE 1:**
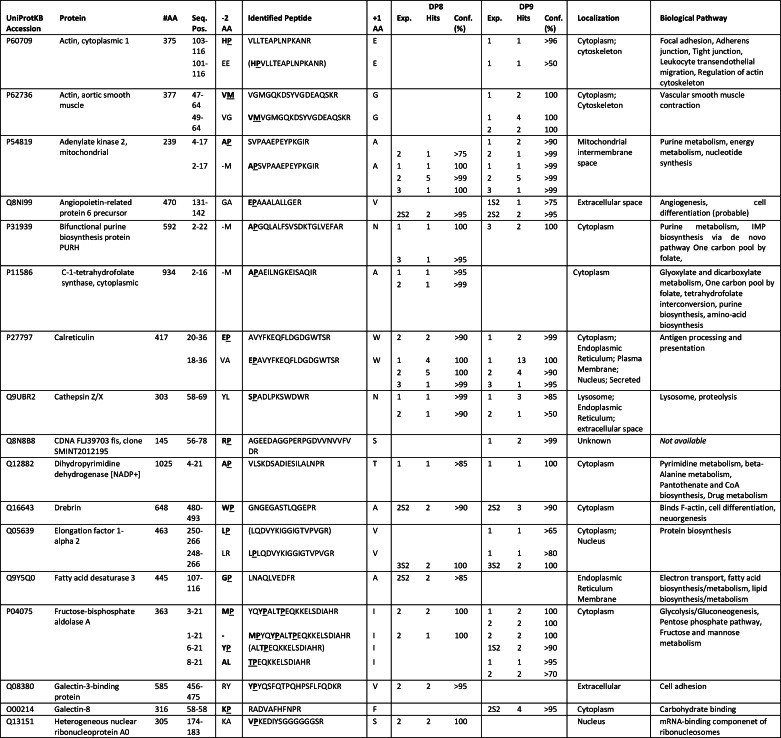

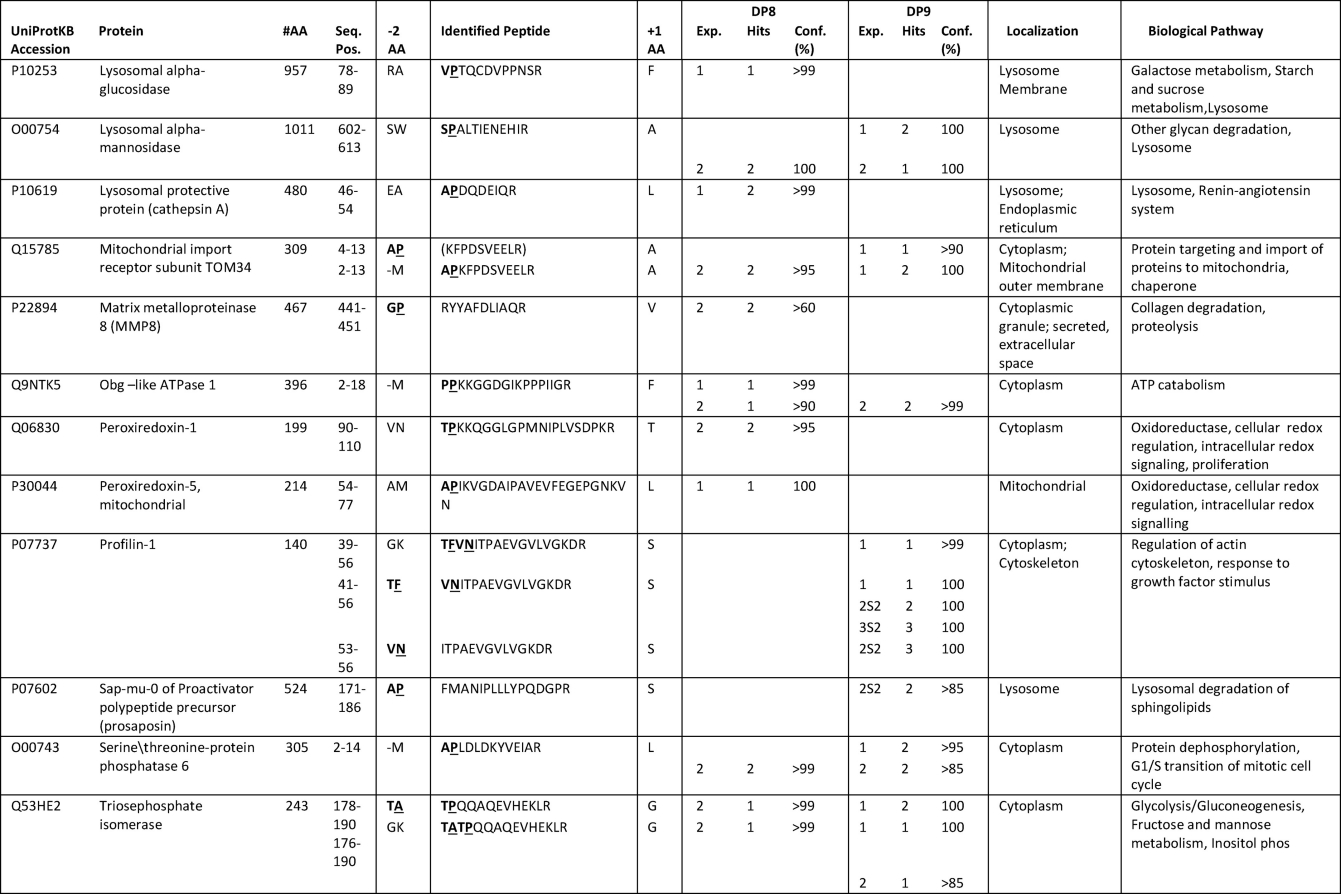
**Candidate DP8 and DP9 substrates** Peptides listed include the following peptides: (i) having a proline N-terminal to the MS/MS-identified peptide so that the peptide may have resulted from DP8/DP9 cleavage; (ii) peptides that contained a Pro in the P1 position; and (iii) peptides differing by two N-terminal dipeptide residues likely to have been removed by DP8/DP9 proteolysis. All individual peptides are listed for DP8 and DP9 in supplemental Tables S2 and S3, respectively. The MS/MS spectra for each of the peptides in the supplemental Tables can be found in the associated supplemental Tables S2 and S3 spectra files. #, number; #AA, number of amino acids in full-length protein; Seq. Pos., position of identified peptide in the full-length protein; −2AA, the two amino acids preceding the identified peptide, of these (−M) indicates initiator methionine; +1 AA, the first amino acid adjacent to the C-terminal end of the MS/MS-identified peptide; Exp, experiment number; Conf. is the confidence determined by Peptide Prophet modeling that the peptide identification was correctly assigned. Peptides in parentheses are those that have a lower confidence in spectra to peptide assignment, but their presence supports the protein being a substrate if identified from a separate high confidence peptide. Bold underlined text indicates the amino acid that DP8/DP9 will cleave at if the identified peptide is a *bona fide* substrate. Where more than one occurrence of a peptide was identified, only the highest confidence was reported in this table. Values for all peptides can be found in supplemental Tables S1 and S2.

##### Identification of Candidate DP8/DP9 Substrates and DP8/DP9 Pathway-affected Proteins by Quantitative Analysis of TAILS Data

Quantitative analysis was only performed on datasets derived from the first two TAILS experiments for both DP8 and DP9 due to the poor recovery of labeled peptides from experiment 3 (data not shown). In total, 37 proteins were identified as potential DP8 substrates or DP8 pathway-affected proteins, and 55 proteins were identified as potential DP9 substrates or DP9 pathway-affected proteins; only 10 of these proteins were common to both DP8 and DP9 (supplemental Table S10). Of these, 17/37 and 10/55 proteins were identified as being candidate substrates of DP8 and DP9, respectively (Table 2), based on analysis of peptide positioning in the UniProtKB annotated protein sequences, *e.g.* peptides located at mature N termini. All other proteins identified (20 for DP8 and 45 for DP9) are listed in supplemental Table S10. Evidence of non-DP8/DP9 N- and C-terminal truncation of proteins was found (supplemental Table S10 and supplemental Figs. S3 and S4), demonstrating that non-DP proteolytic pathways are altered by increasing DP8/DP9 activity. Interestingly, approximately half of the proteins identified for DP8 are mitochondrial, potentially occurring during mitochondrial turnover (Table 2 and supplemental Table S10). Functional annotation and pathway mapping revealed that many of the identified proteins play key roles in carbohydrate, nucleotide, and protein metabolism (supplemental Tables S8 and S9).

**TABLE 2:**
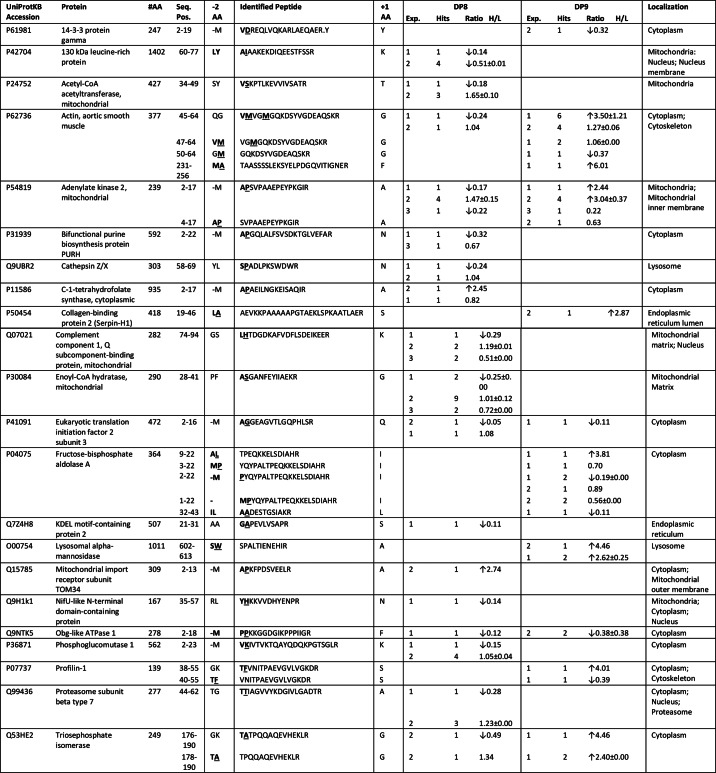
**Candidate DP8 and DP9 substrates identified by quantitative analysis of TAILS data** Peptides were identified as significantly increased or decreased in the active DP8 EGFP or DP9-EGFP SKOV3 cells compared with the catalytically inactive forms. Individual peptides identified by MS/MS can be found for DP8 and DP9 in supplemental Tables S4 and S5, respectively. The MS/MS spectra for each of these peptides can be found in the associated supplemental Tables S4 and S5 spectra files. #, number; #AA, number of amino acids in full-length protein; Seq. Pos., position of identified peptide in the full-length protein; −2AA, the two amino acids preceding the identified peptide, of these (−M) indicates initiator methionine; +1AA, the first amino acid adjacent to the C-terminal end of the MS/MS-identified peptide; Exp., experiment number; Hits, number of spectra positively identified for each peptide; Ratio H/L, the isotopic heavy/light ratio. Ratios are expressed as means ± S.D. Bold underlined text indicates the amino acid that DP8/DP9 will cleave at if the identified peptide is a *bona fide* substrate. Arrows (↓/↑) indicate whether the presence of peptide was significantly increased or decreased.

##### In Vivo Substrate Specificity of DP8 and DP9

**FIGURE 4.**
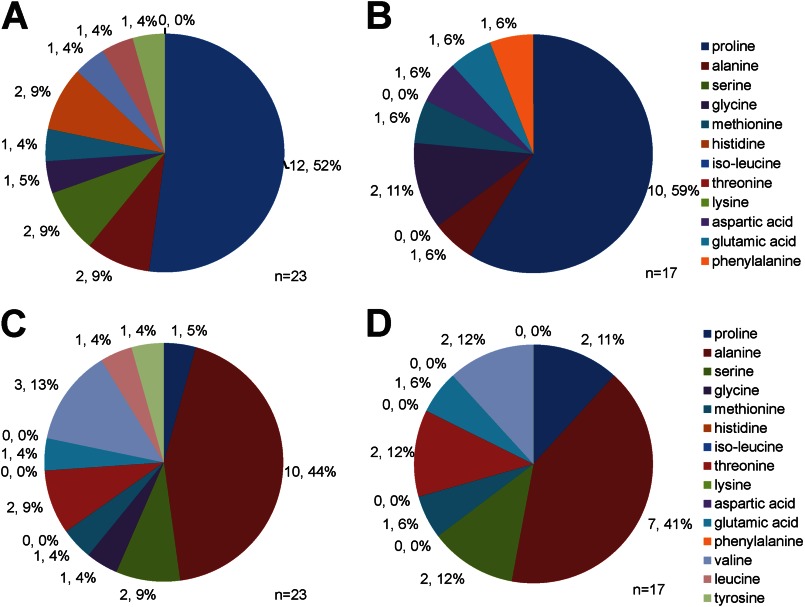
**Frequency distribution of residues in P1 and P2 position of DP8 and DP9 candidate substrates.** The frequency distribution of amino acids in the P1 (*A* and *B*) and P2 (*C* and *D*) position of candidate substrates identified in Table 3 are given for DP8 (*A* and *C*) and DP9 (*B* and *D*). The exact number of peptides in which each residue is found in the P1 or P2 position is given along with the percentage relating to frequency distribution.

Fig. 4 displays the frequency of residues in the P1 and P2 position of all DP8 and DP9 candidate substrates identified in this study. As expected, the majority of candidate substrates contain a Pro in the P1 position (Fig. 4, *A* and *B*) followed by an Ala in the P2 position (Fig. 4, *C* and *D*). The frequency of residues in the P1 and P2 position for the remaining candidate substrates was variable for both DP8 and DP9 (Fig. 4).

**FIGURE 5.**
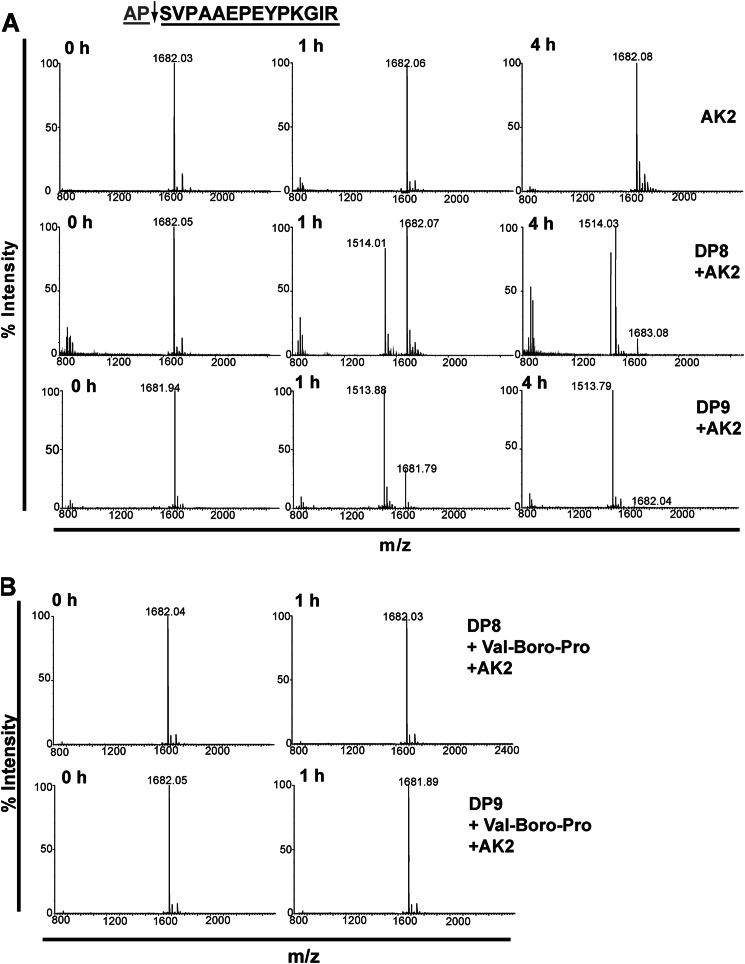
**DP8 and DP9 cleave the N-terminal peptide of adenylate kinase 2 *in vitro*.** The N-terminal peptide of mature adenylate kinase 2 is displayed. The *arrow* indicates the site of DP8/DP9 proteolysis with the *gray text* indicating the dipeptide that is removed following DP8/DP9 proteolysis. *A,* 10 μm of the adenylate kinase 2 peptide was incubated alone or with 1.7 milliunits of active purified recombinant DP8 or DP9. Samples were collected and stopped with 1% TFA (v/v) final at 0, 1, and 4 h. MS spectra were acquired for noncleaved (*m*/*z*) and DP8/DP9-cleaved (*m*/*z*) peptides with a mass accuracy of 0.01 to 0.05% error. Theoretical masses of noncleaved and cleaved peptides are 1681.89 and 1512.79 Da, respectively. *B,* specificity of cleavage by DP8/DP9 of these peptides was confirmed by performing catalysis reactions in the presence of 10 μm of the nonselective DP inhibitor, Val-Boro-Pro (PT-100/Talabostat). Displayed spectra are representatives from three independent cleavage experiments. *AK2*, adenylate kinase 2.

**FIGURE 6.**
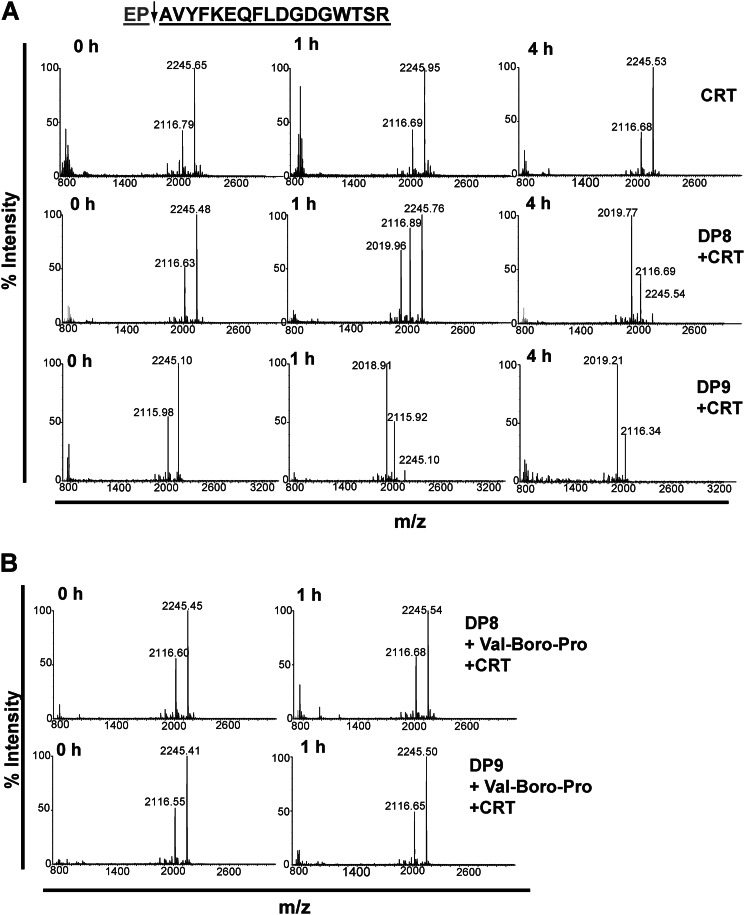
**DP8 and DP9 cleave the N-terminal peptide of calreticulin *in vitro*.** The N-terminal peptide of mature calreticulin is displayed. The *arrow* indicates the site of DP8/DP9 proteolysis, and the *gray text* indicates the dipeptide that is removed following DP8/DP9 proteolysis. *A,* 10 μm of the calreticulin was incubated alone or with 1.7 milliunits of active and purified recombinant DP8 or DP9. Samples were collected and stopped with 1% TFA (v/v) final at 0, 1, and 4 h. MS spectra were acquired for noncleaved (*m*/*z*) and DP8/DP9-cleaved (*m*/*z*) peptides with a mass accuracy of 0.01 to 0.05% error. Theoretical masses of noncleaved and cleaved peptides were 2245.45 and 2017.95 Da, respectively. *B,* specificity of cleavage by DP8/DP9 of these peptides was confirmed by performing catalysis reactions in the presence of 10 μm of the nonselective DP inhibitor, Val-Boro-Pro (PT-100/Talabostat). Displayed spectra are representatives from three independent cleavage experiments. *CRT,* calreticulin.

DP8 and DP9 cleave *in vitro* the N termini of adenylate kinase 2, calreticulin, and other peptides. *In vitro* validation of DP8/DP9 cleavage was performed for 14 candidate substrates, including the two most abundant substrates, adenylate kinase 2 and calreticulin (Table 3, Figs. 5 and 6, and supplemental Fig. S5). In total, cleavage of 9/14 substrates by both DP8 and DP9 was confirmed. No unique cleavage was identified for DP8 or DP9 demonstrating similar enzyme specificity and large substrate overlap between these enzymes.

**TABLE 3:**
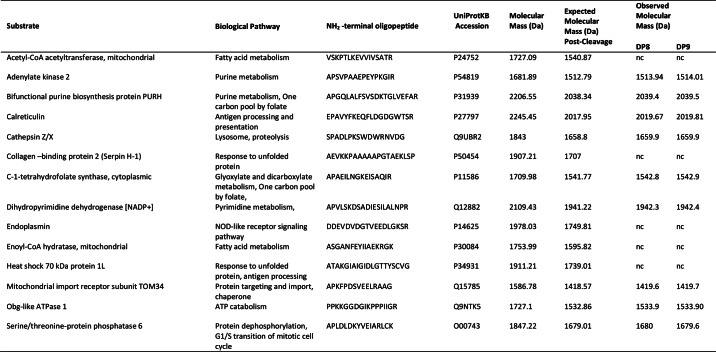
**Confirmed cleavage of DP8 and DP9 substrates** For each substrate, 10 μm of the N-terminal oligopeptide was incubated with 1.7 milliunits of active, purified recombinant DP8 or DP9. Observed molecular mass (Da) was determined from MS spectra acquired 24 h after incubation, nc means not cleaved.

**FIGURE 7.**
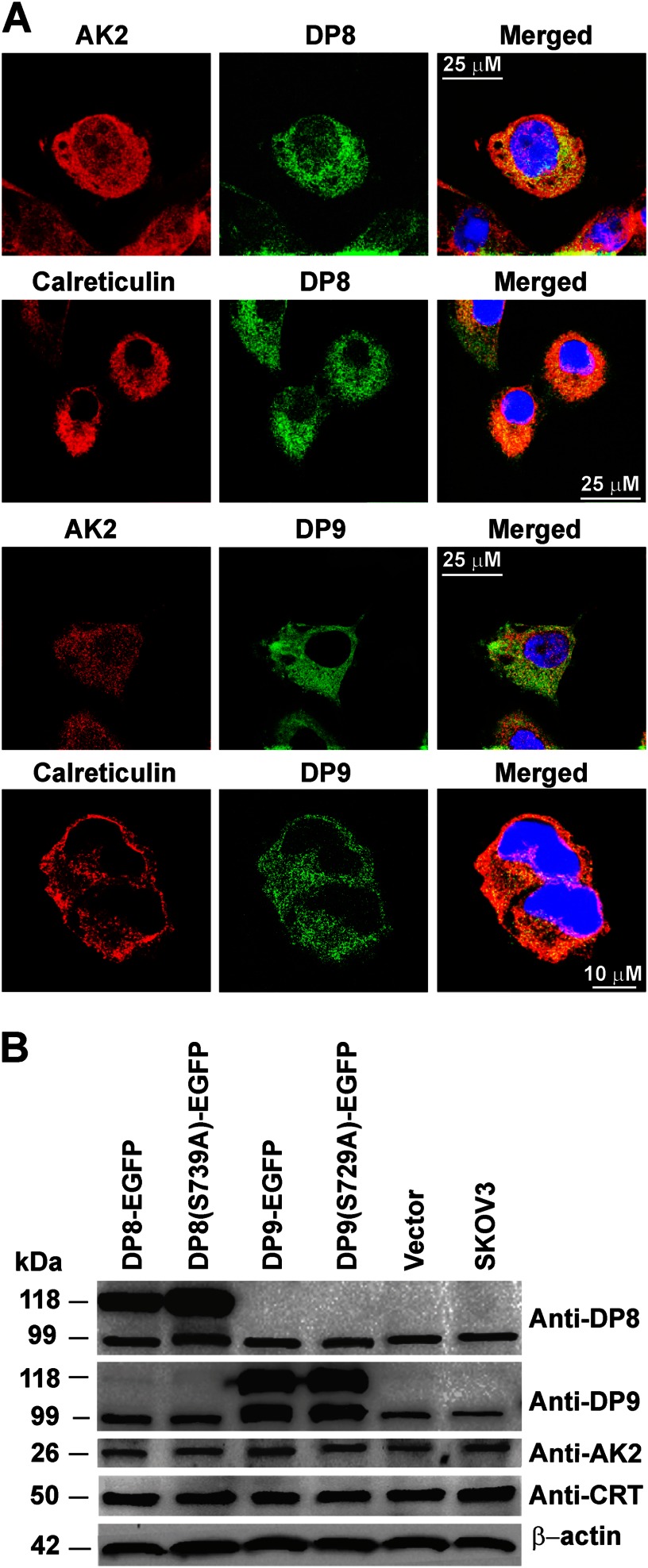
**Expression and co-localization of adenylate kinase 2 and calreticulin with DP8 and DP9 in SKOV3 cells.** *A,* DP8-EGFP- and DP9-EGFP SKOV3-expressing cells lines were analyzed by confocal microscopy and immunofluorescence using anti-calreticulin (1:50) and anti-adenylate kinase 2 (1:50) polyclonal antibodies. As labeled, *red panels* display adenylate kinase 2 or calreticulin, and *green panels* display DP8-EGFP or DP9-EGFP expression. Merged images are shown in the *far right-hand panels. B,* cell lysates (25 μg) from DP8-EGFP, DP8(S739A)-EGFP, DP9-EGFP, DP9(S729A)-EGFP, vector-transfected, and nontransfected SKOV3 cells were analyzed by 10% (w/v) SDS-PAGE and immunoblotting using DP8 (1:5000), anti DP9 (1:5000), calreticulin (1:10,000), adenylate kinase 2(1:5000), and β-actin (1:10,000) as a loading control. *AK2,* adenylate kinase 2; *CRT,* calreticulin.

In comparison with DP8, DP9 more readily cleaved the N-terminal peptide of adenylate kinase 2 (Fig. 5*A*) demonstrating a probable higher affinity of recombinant DP9 for this substrate *in vitro*. Recombinant DP8 cleaved N-terminal peptides of adenylate kinase 2 and calreticulin with similar kinetics, whereas DP9 displayed a faster rate of cleavage for the calreticulin N-terminal peptide compared with adenylate kinase 2 (Figs. 5*A* and 6*A*). Specificity of DP8/DP9 cleavage was confirmed by performing peptide catalysis reactions in the presence of the nonselective DP inhibitor, Val-Boro-Pro (PT-100/Talabostat) (Figs. 5*B* and 6*B*). Co-localization of DP8- and DP9-EGFP with both adenylate kinase 2 and calreticulin was demonstrated by confocal microscopy (Fig. 7*A*) confirming physical proximity between protease and substrate. No difference in the localization of either adenylate kinase 2 or calreticulin was detected between the active DP8- and DP9-EGFP cell lines compared with the catalytically inactive DP8(S739A)-EGFP and DP9(S729A)-EGFP cells (data not shown). Immunoblots demonstrated that full-length adenylate kinase 2 and calreticulin are not altered in their expression levels in the DP8- or DP9-EGFP SKOV3 cells lines (Fig. 7*B*) indicating that DP8/DP9 proteolysis is unlikely to alter the stability of these two proteins.

## DISCUSSION

This is the first cytosol-wide analysis of proteome regulation by DP8 and DP9. In contrast to other studies focused on individual substrates in *in vitro* assays (18, 20, 21), we have utilized TAILS (22), an N-terminally focused negative selection proteomics approach for the first time on a cytoplasmic proteome, to identify *in vivo* DP8 and DP9 substrates. In this study, stable transfected SKOV3 cells were used to identify 23 and 17 candidate substrates of DP8 and DP9 respectively; 14 of these were selected for *in vitro* validation. Adenylate kinase 2, calreticulin, and seven other substrates were validated as DP8/DP9 substrates. This quantitative analysis identified additional proteins involved in pathways regulated or affected by overexpression of DP8 (20 proteins) and DP9 (45 proteins) enzyme activity. Many of the proteins identified by quantitative analysis and those confirmed as DP8/DP9 substrates are involved in regulating cellular metabolism and energy homeostasis. Thus, this study for the first time reveals potential roles for DP8 and DP9 in cellular metabolic pathways, including glycolysis, gluconeogenesis, fatty acid metabolism, and nucleotide metabolism/biosynthesis. In addition, the involvement of calreticulin in the antigen processing and presentation is consistent with a role for DP9 proteolysis in preventing cell surface presentation of the major histocompatibility complex class I antigen (21).

Many of the candidate substrates in Table 3 were identified only from peptides with the potential for DP8/DP9 cleavage, with little to no detection of the cleaved peptides. This is likely due to the cleaved peptides being of low abundance or may indicate their rapid degradation following DP8/DP9 truncation. For some targets, contradictory isotopic heavy/light ratios were identified, and discrepancies were observed with ratios between different datasets. These contradictory ratios are probably due to the endogenous enzyme levels of DP8 and DP9 present in all our cell lines. It is likely that background endogenous DP8/DP9 proteolysis also contributes to the identification of overlapping substrates of DP8 and DP9.

### 

#### 

##### In Vivo Substrate Specificity of DP8 and DP9

Candidate substrates identified in this study confirm *in vivo* the *in vitro* work of others demonstrating a preference for both DP8 and DP9 to cleave the post-prolyl bond (33–35), with a strong preference for substrates with an Ala in the P2 position followed by Val, Thr, Ser, or Pro residues. Our findings also reveal the potential for DP8/DP9 cleavage to occur after nontypical residues in the P1 position; however, all validated substrates contained a Pro in P1 (Table 3). In addition an *in vivo* preference was observed for the potential cleavage of dipeptides from substrates with an Ala, Lys, Val, Thr, or Gly residue in what would be the P1′ position. Roles for P1′ and P2′ residues in DP8 substrate specificity have been previously suggested following observations of DP8 *in vitro* cleavage of chemokines where DP8 kinetically favored cleavage of chemokines containing Ser in P2′ and Leu and Val in P1′ (20).

##### Identification of Noncytoplasmic Substrates

A number of DP8/DP9 substrates and pathway-affected proteins identified in this study are known to be localized in intracellular organelles. Approximately half of the identified DP8 substrate candidates are mature mitochondrial proteins for which a mitochondrial targeting sequence is absent (*e.g.* adenylate kinase 2) or has been removed. These findings suggest that in the DP8 cell lines, there may be increased mitochondrial turnover/degradation resulting in leakage of proteins from autophagosomes (as occurs for acetyl-CoA acetyltransferase (36)) suggesting that DP8 may play a role in mitochondrial homeostasis. Although it may be possible that some nuclear, mitochondrial, and other noncytoplasmic proteins are released upon cell lysis and thus accessible to DP8/DP9 cleavage, it is thought that this was not likely due to the gentle method of cell lysis used in this study, the rapid inclusion of protease inhibitors during lysis, and to the differences in localization of proteins between the DP8 and DP9 datasets.

##### Adenylate Kinase 2 and Calreticulin as Natural Endogenous DP8 and DP9 Substrates

Co-localization *in vivo* of a protease with its substrate is required for cleavage. By demonstrating this by co-localization studies, it supports the *in vivo* relevance of adenylate kinase 2 and calreticulin as natural substrates of DP8 and DP9. Although determination of the biological importance of the cleavage of adenylate kinase 2 and calreticulin by DP8/DP9 is difficult due to the pleiotropic effects of both adenylate kinase 2 and calreticulin (37), evidence for a potential biological role for DP8/DP9 processing in adenylate kinase 2 and calreticulin function exists in the literature. DP8 and DP9 may play a role in the post-translational modification of adenylate kinase 2, prior to its mitochondrial import, to produce a known variant lacking the N-terminal “MAP” sequence, which has a 2-fold higher activity than the major variant with the Ala-Pro dipeptide intact (38). DP8/DP9 proteolysis of adenylate kinase 2 may also occur following its apoptosis-induced release from the mitochondria, potentially altering its binding affinity to Fas-associated protein with death domain and activation of apoptosis via a novel pathway (39). Calreticulin is known to be retrotranslocated from the endoplasmic reticulum lumen to the cytoplasmic space after removal of its N-terminal signal peptide (residues 1–17) (40). In the cytosol, calreticulin has been shown to undergo post-translational arginylation of the exposed N-terminal aspartic acid residue (41, 42). Under stress conditions, arginylated calreticulin associates with stress granules in a calcium-dependent manner (41). Such a modification would make the N termini of cytosolic calreticulin inaccessible to DP8/DP9; however, under the basal conditions used in our study, no arginylated N-terminal peptides of calreticulin were identified in our datasets (data not shown). Indeed, we did identify DP8/DP9-cleaved N-terminal peptides of calreticulin. Potentially, DP8/DP9 are involved in regulating the function and subcellular localization of cytoplasmic calreticulin in response to stress. In our study, under basal conditions, no change in calreticulin localization was observed following overexpression of active or catalytically inactive DP8 and DP9; however, under conditions of stress this may be altered.

The N-end rule specifies that the stability of a protein can be affected by the identity of its N-terminal residue (43). Although it is thought possible that DP8/DP9 may be involved in degradation of proline-containing proteins by affecting their stability upon N-terminal cleavage, no increase or decrease in the expression of full-length adenylate kinase 2 or calreticulin was observed in this study following overexpression of DP8 or DP9 (Fig. 7*B*).

In conclusion, this study has identified and validated a number of biologically important candidate *in vivo* substrates for both DP8 and DP9, both previously enigmatic proteases with only one *in vivo* substrate known. Furthermore, this work has highlighted roles for both of these proteases in cellular energy metabolism and homeostasis. Importantly, adenylate kinase 2 and calreticulin were identified and validated as substrates of both DP8 and DP9. Adenylate kinase 2 plays an important role in maintaining cellular energy homeostasis, and thus DP8/DP9 proteolysis may contribute to regulating cellular energy homeostasis through adenylate kinase 2. TAILS is a powerful proteomic approach for the discovery of protease substrates. The recent use of TAILS in *in vivo* analysis of tissue samples opens the possibility for similar analysis of DPs in the future. For the first time, we have applied TAILS to a cytoplasmic proteome and found the approach to be particularly well suited to the discovery of N-terminal substrates of the exopeptidases DP8 and DP9. This proteomic investigation has identified some unique but largely overlapping roles and substrates for DP8 and DP9, thus paving the way for ongoing investigations into the fundamental roles of DP8 and DP9 in cellular metabolism and homeostasis.

## Footnotes

7 The abbreviations used are:

DP: dipeptidyl peptidase
EGFP: enhanced green fluorescent protein
IPI: International Protein Index
TAILS: terminal amine isotopic labeling of substrates.
